# 

**Published:** 1886-10-30

**Authors:** 


PRICE ONE PENNY.

No. 5, Vol. I.
October 30th, 1886.

Per Annum, 6s. 6d., post free.
Monthly Parts, 6d.; post free, 7½d.
Ditto, per Ann., 7s. 6d., post free.

